# John B. Lillard & Co.

**Published:** 1881-08-20

**Authors:** 


JNO. B. LILLARD & CO., dealers in Instruments, Trusses, etc., Nashville, Tenn. We are in receipt of Hypodermic Syringes and Chemical Thermometers from this fine establishment, recently instituted at Nashville. As a place to secure Instruments in the Surgical line, we feel safe in recommending this house as worthy the support and confidence of the Profession. In our dealings with this House we have found the Proprietors prompt, business-like and accommodating.

